# Fabrication and characterization of a calcium-induced mung bean protein nanofibrils emulsion gel with tailored rheology for 3D food printing

**DOI:** 10.1016/j.fochx.2026.104246

**Published:** 2026-07-25

**Authors:** Yupeng Qi, Yuhe Wang, Chunhong Liu, Dongmei Li, Zhibiao Feng, Bin Jiang, Jing Xu

**Affiliations:** a College of Chemistry and Molecular Engineering, Northeast Agricultural University, Harbin 150030, China; b Key Laboratory of soybean Biology of Ministry of Education China, Northeast Agricultural University, Harbin, China; c Heilongjiang Green Food Science Research Institute, Harbin, China

**Keywords:** Mung bean protein isolate, Nanofibrils, Ca^2+^, Emulsion gel, 3D printing

## Abstract

Protein nanofibrils (PNFs) show superior functional properties over native proteins, making them promising building blocks for advanced food materials. In this study, MPI emulsions display inadequate gelation properties, which cannot satisfy the printability requirements for 3D food fabrication. Mung bean protein isolate nanofibrils (MPNFs) were fabricated by an acid-thermal method (pH 2.0, 90 °C, 11 h), and calcium-induced emulsion gels were developed for extrusion-based 3D food printing. TEM confirmed the formation of worm-like MPNFs. MPNFs-stabilized emulsions formed self-supporting gels with 100 mM Ca^2+^ upon heating. The optimized emulsion gel exhibited a uniform microstructure, high water-holding capacity (93.29%), and excellent rheological properties (G′ ≈ 850 Pa, shear-thinning behavior and successful high-fidelity printing). It enabled high-fidelity 3D printing without collapse. This work provides a feasible strategy for designing plant protein nanofibril-based emulsion gels as edible inks for personalized food manufacturing.

## 1 Introduction

One of the key challenges in extrusion-based 3D food printing is the lack of plant-based inks with both shear-thinning behavior and rapid structural recovery. Emulsion gels are promising candidates, but whether mung bean protein nanofibrils (MPNFs) can form such inks remains unexplored. Emulsion gels, which combine the structural integrity of a gel matrix with the encapsulation capacity of emulsion droplets, have emerged as promising ink candidates for 3D food printing (Jantrawut et al., 2023; Keerthana, Anukiruthika, Moses, & Anandharamakrishnan, 2020). These soft-solid materials possess tunable viscoelastic properties that can be tailored to meet the stringent rheological demands of extrusion-based printing (Zhi et al., 2023). Moreover, emulsion gels can serve as carriers for lipophilic bioactive compounds (Zhong et al., 2020), offering opportunities for designing functional foods targeting specific dietary needs (Shi, Chen, & Meng, 2023).

Recent advances have demonstrated the feasibility of using emulsion gels as inks for extrusion-based 3D food printing. For instance, protein-stabilized emulsion gels have been successfully printed to create customized structures with tailored textures and nutritional profiles (Wang et al., 2023a; Yellapantula, Dayananda, Bhandari, & Prakash, 2026). Polysaccharide-based emulsion gels have also been explored for their shear-thinning and recovery properties, which are critical for layer-by-layer deposition (Zhou, Ge, Ma, Zhang, & Wang, 2025). However, most existing studies rely on animal-derived proteins (e.g., whey, egg white) or synthetic polysaccharides. Plant-protein-based emulsion gels, especially those designed for 3D printing, remain underexplored. In particular, the potential of mung bean protein-a sustainable, hypoallergenic plant protein-has not been investigated in this context.

Among plant proteins, mung bean protein isolate (MPI) has garnered increasing attention due to its high nutritional quality (Hou et al., 2019), favorable amino acid profile (Tarahi, Abdolalizadeh, & Hedayati, 2024), and low allergenicity (Chakravorty, Badwaik, & Das, 2026; Guo et al., 2026; Hashemi, Assadpour, Wang, & Jafari, 2025). However, the native functional properties of MPI, such as its emulsifying capacity and gelation behavior, often fall short of the requirements for constructing robust emulsion gels suitable for 3D printing (Niu, Zhang, Mujumdar, & Li, 2026; Qiao et al., 2025). Specifically, MPI-stabilized emulsions typically require high protein concentrations or extensive cross-linking to form self-supporting gels, limiting their application in low-fat or cost-effective formulations (Cui, Guo, & Meng, 2023; Liu, Zhang, Bhandari, & Wang, 2017).

A promising strategy to overcome these limitations lies in the transformation of globular proteins into protein nanofibrils (PNFs) through acid-heat induced self-assembly (Pan et al., 2026). PNFs, characterized by their high aspect ratio, enhanced surface hydrophobicity, and ordered *β*-sheet-rich structures, exhibit superior interfacial adsorption and gel network-forming capabilities compared to their native counterparts (Wang et al., 2020; Xu et al., 2025). These attributes make PNFs particularly attractive as building blocks for designing emulsion gels with tailored microstructures and rheological properties (Qi et al., 2024).

While the formation and applications of PNFs from animal proteins (whey protein, egg white protein) have been widely studied (Shen, Wang, & Li, 2020; Tarahi et al., 2024), the study of plant-derived PNFs, especially those from mung bean protein isolate (MPI), remains largely in its infancy (Hou et al., 2019). Regarding mung bean, several recent studies have demonstrated the feasibility of fabricating MPI fibrils and explored their potential applications. Qi et al. (Qi et al., 2024) reported the successful formation of MPNFs via acid-heat treatment and investigated their calcium-induced hydrogelation, demonstrating that MPNFs can form hydrogels with tunable mechanical properties. Herneke et al. (Herneke, Karkehabadi, Lu, Lendel, & Langton, 2023) examined the effect of pH on MPNFs morphology and their ability to stabilize foams, revealing that fibril morphology significantly influences interfacial behavior. Collectively, these studies have established the fundamental feasibility of producing MPNFs and highlighted their potential in hydrogels, foams, and bioactive delivery systems.

Therefore, the key scientific questions to be addressed are whether MPNFs can form emulsion gels,and whether such MPNFs-based emulsion gels possess the rheological properties required for 3D food printing—specifically, how their fibrillar architecture translates into emulsion gel microstructure, how this microstructure governs the macroscopic rheological and textural properties, and ultimately, how these properties determine 3D printability (Schutyser, Houlder, de Wit, Buijsse, & Alting, 2018).

Herein, we hypothesize that the acid-heat induced fibrillation of MPI into worm-like nanofibrils (MPNFs) fundamentally enhances its functionality, enabling the fabrication of a robust calcium-induced emulsion gel with precisely tunable rheology that meets the stringent requirements of 3D printing. To test this hypothesis, we systematically: (i) fabricated and characterized MPNFs, including their morphological evolution during self-assembly; (ii) engineered MPNFs-based emulsion gels by modulating protein concentration, oil phase fraction, and Ca^2+^ concentration, with MPI-based gels as controls; (iii) established structure-function relationships by correlating the emulsion gel microstructure (CLSM, particle size) with its hydration properties (WHC, LF-NMR), mechanical behavior (rheology, texture profile), and stability; and (iv) evaluated the 3D printability of the optimized formulations by constructing complex geometries. This work not only valorizes an underutilized plant protein resource but also provides a mechanistic understanding of how protein fibrillation can be harnessed to design next-generation edible inks for personalized food manufacturing.

## 2 Materials and methods

### 2.1 Materials

Mung bean protein powder (protein content 82.3% on dry basis, determined by Kjeldahl, N × 6.25) was obtained from Hadar Starch & Starch Co. Ltd. (Harbin, China). Soybean oil was purchased from Jiusan Grain and Oil Industry Group Co., Ltd. (Beijing, China). Nile Red, Nile Blue, and all other chemicals and reagents used were of analytical grade and were bought from Aladdin Biochemical Technology Co. Ltd. (Shanghai, China).

### 2.2 Preparation and characterization of MPNFs

#### 2.2.1 Preparation of MPI

Mung bean protein isolate (MPI) was extracted following the method of Wang et al. (Wang et al., 2020) with modifications. Mung bean protein powder was dispersed in ultrapure water (5%, w/v), and the pH was adjusted to 9.0 using 0.1 mol/L NaOH. The dispersion was stirred at 40 °C for 30 min at 300 rpm using a magnetic stirrer to ensure complete hydration. The supernatant was collected after centrifugation at 3000×*g* for 10 min, and its pH was adjusted to 4.5 (the isoelectric point of MPI) using 0.1 mol/L HCl. The precipitate obtained after centrifugation (3000×*g*, 10 min) was collected, freeze-dried, and stored at 4 °C until use. The protein content of the resulting MPI was 91.6 ± 0.8% (Kjeldahl, N × 6.25).

#### 2.2.2 Preparation of MPNFs

MPNFs were prepared according to Qi et al. (Qi et al., 2024) with slight modifications. MPI powder was dispersed in ultrapure water (5%, w/v), and the pH was adjusted to 2.0 using 6 mol/L HCl. The solution was stirred at 300 rpm for 30 min at 25 °C, followed by centrifugation at 11,000×*g* for 15 min at 4 °C to remove insoluble aggregates. The supernatant was filtered through a 0.45 μm cellulose acetate membrane, and the filtrate was incubated in a water bath at 90 °C with continuous shaking (220 rpm) for 11 h to induce fibrillation. The resulting MPNFs solution (pH 2.0) was used immediately or stored at 4 °C for no more than 3 days. The final protein concentration after fibrillation was 4.8 ± 0.1% (w/v), as determined by the Biuret method. The average length and diameter of MPNFs were 1.2 ± 0.4 μm and 8.5 ± 2.1 nm, respectively, as measured from TEM images (*n* = 100) using ImageJ software. No dialysis or neutralization was performed to avoid disrupting the fibrillar structure (Xu, Liu, et al., 2025).

#### 2.2.3 Transmission electron microscopy (TEM)

TEM settings for MPNFs were slightly modified according to the method by Zhang et al. (Zhang et al., 2024). The MPNFs solution was diluted 40 times using HCl solution (pH 2.0) and then ultrafiltered with a cutoff of 100 kDa at 3000×*g* for 15 min. The final dilution was approximately 10-fold. The solution was placed on a copper grid on a carbon film for drying and adsorption for 15 min. Then, the sample was negatively stained with 2% uranyl acetate. The microscopic morphology of the MPNFs was observed using a transmission electron microscope (HT7800, Hitachi, Japan).

### 2.3 Preparation and characterization of MPNFs emulsion gels

#### 2.3.1 Preparation of emulsion gels

Emulsion gels were prepared following the method of Wei et al. (Wei et al., 2025) with modifications. The MPNFs solution (aqueous phase, 3%, 4%, 5%, w/v) was mixed with soybean oil (oil phase) at the desired oil phase fractions (0.25, 0.5, or 0.75, v/v). CaCl₂ was added to the mixture at final concentrations of 0, 50, or 100 mM to promote gelation through ionic cross-linking. The mixture was immediately homogenized at 7000 rpm for 2 min using a high-shear homogenizer (T18, IKA, Germany) to form a coarse emulsion. The resulting emulsion was transferred to a sealed glass vial and heated in a water bath at 80 °C for 30 min to promote gel network formation. After heating, the samples were cooled to 25 °C and stored at 4 °C for 24 h to allow complete gel maturation before characterization. The emulsion gels were designated as X–Y–Z, where X represents the MPNFs concentration (%, w/v), Y represents the oil phase fraction (v/v), and Z represents the CaCl₂ concentration (mM). For example, 5%-0.5–100 denotes an emulsion gel prepared with 5% (w/v) MPNFs, 0.5 oil phase fraction, and 100 mM CaCl₂.

Emulsion gels based on MPI (3%, 4%, 5%, w/v) were prepared using the same procedure as the MPNFs emulsion gels. The MPI-based emulsion gel was labeled as 5%P-0.5-100, where “P” stands for MPI.

#### 2.3.2 Storage stability

The emulsion gels were stored based on a previously published method (Kan et al., 2023) with slight modifications. The gel samples were stored at room temperature, and changes were observed on days 1, 30, and 60. Photos were taken for observation and comparison.

#### 2.3.3 Determination of WHC

The method employed was based on the approach of Zeng et al. (Zeng et al., 2025) with slight modifications. The emulsion gel was centrifuged at 8000×*g* for 30 min at 4 °C. After centrifugation, the precipitated water was carefully removed from the gel. The blank centrifuge tube, centrifuge tube before centrifugation, and centrifuge tube after water absorption were weighed. The WHC was calculated as formula (1):

(1) $$\mathrm{WHC}\left(\%\right)=\frac{M_{2}-M_{0}}{M_{1}-M_{0}}\times 100\%$$

*M*_*0*_, *M*_*1*_, and *M*_*2*_ represent the weight of the empty centrifuge tube, weight of the centrifuge tube before centrifugation, and weight of the centrifuge tube after water absorption, respectively.

#### 2.3.4 Low-field nuclear magnetic resonance (LF-NMR)

A MesoMR23-060H-I NMR spectrometer (MesoMR23-060H-I, Suzhou Newmai Analytical Instrument Co., Ltd.) was used to analyze the proton relaxation time of the samples. T_2_ was determined at a frequency field of 20 MHz. A CPMG pulse sequence was used, with the following pulse parameters: waiting time (T_W_), 1500 ms; interval between two peaks (T_E_), 0.5 ms; number of identified points (NECH), 1500; interval between points (S_W_), 100 kHz; and number of scans (N_S_), 8. T_2_ distribution curves were established using logarithmic coordinates (Chen et al., 2023).

#### 2.3.5 Rheological properties

The viscoelasticity of the emulsion gels was measured using a frequency scanning rheometer (HAAKE MARS60, Thermo Fisher Scientific Shier Technology Co., Ltd.) following the method proposed by Dai et al. (Dai et al., 2025). The sample was placed between parallel plates, with a scanning frequency of 0.1–10 Hz. The storage (G′) and loss (*G*″) moduli were recorded as frequency functions.

The apparent viscosity of the gels was also quantified using a rheometer. The sample was subjected to a shear rate of 1–100 s^−1^ between parallel plates. All experiments were performed at a temperature of 25 °C.

#### 2.3.6 Texture analysis

The textural properties of the composite hydrogels were analyzed using a texture analyzer (TA.XT Plus C, Stable Micro System, UK) with the texture profile analysis mode. A P/0.5 probe was selected to test the emulsion gels for three compression cycles. The probe entry distance was set to 10 mm, the pre-test, mid-determination probe, and post-determination probe speeds were 1 mm/s, the hold time was 5 s, and the trigger force was Auto-5 g. Measurements were performed at 25 °C (Niu et al., 2026).

#### 2.3.7 Confocal laser scanning microscopy (CLSM)

A mixed fluorescent dye was prepared by combining Nile red and Nile blue at a concentration of 1% each using anhydrous ethanol as the solvent. The resulting dye was used to stain the emulsion gels on a glass slide. To observe the sample and collect images, a laser confocal microscope (FV3000, Japan Olympus Company) was utilized. Nile red and Nile blue were excited at wavelengths of 488 and 633 nm, respectively (Wang et al., 2023b).

#### 2.3.8 Particle size determination

An appropriate amount of the emulsion gels was dispersed in deionized water, 2% sodium dodecyl sulfate solution, and mixture of 2% sodium dodecyl sulfate and 1% mercaptoethanol. Particle size distribution was analyzed using a laser particle size analyzer (S3500, USA Microtrac Company), and the mean diameter was measured (d_4,3_), assuming a refractive index of 1.33 for the aqueous phase and 1.46 for soya oil (Ye Zhang et al., 2024).

### 2.4 3D printing

The 3D printability of emulsion gels was evaluated using an extrusion-based 3D food printer (Wiiboox Sweetin, Nanjing, China) equipped with a 20 mL stainless steel syringe and a conical plastic nozzle (inner diameter: 1.2 mm). The printing parameters were as follows: nozzle height, 1.5 mm; extrusion pressure, 0.2–0.3 MPa; printing speed, 20 mm/s; layer height, 1.0 mm; infill density, 100%. The printing path was generated using Slic3r software. Emulsion gels were loaded into the syringe and printed at 25 °C to fabricate 3D models including pentagrams (30 × 30 × 5 mm), cubes (20 × 20 × 20 mm), and a turtle model (60 × 40 × 15 mm). After printing, the structures were photographed immediately and after 30 min to assess shape retention and stability (Wang et al., 2023b).

The 3D printed cubes (designed edge length: 20.0 mm) were measured immediately after printing and after storage at room temperature (25 °C) for 30 min. The length width and height were measured using a stainless steel digital caliper (accuracy: 0.1 mm). Three independent cubes were printed for each formulation, and each cube was measured three times at different positions (left edge, center, right edge). The average length was calculated.

### 2.5 Statistical analysis

All treatments were replicated at least three times. Statistical analyses were performed using Origin8.0 and DPS7.05. Data presented in tables and figures are expressed as means with standard deviations (error bars). Significant differences between sample means were identified at *p* < 0.05.

## 3 Results and discussion

### 3.1 Microscopic morphology analysis of MPNFs self-assembly formation

**Fig. 1:**
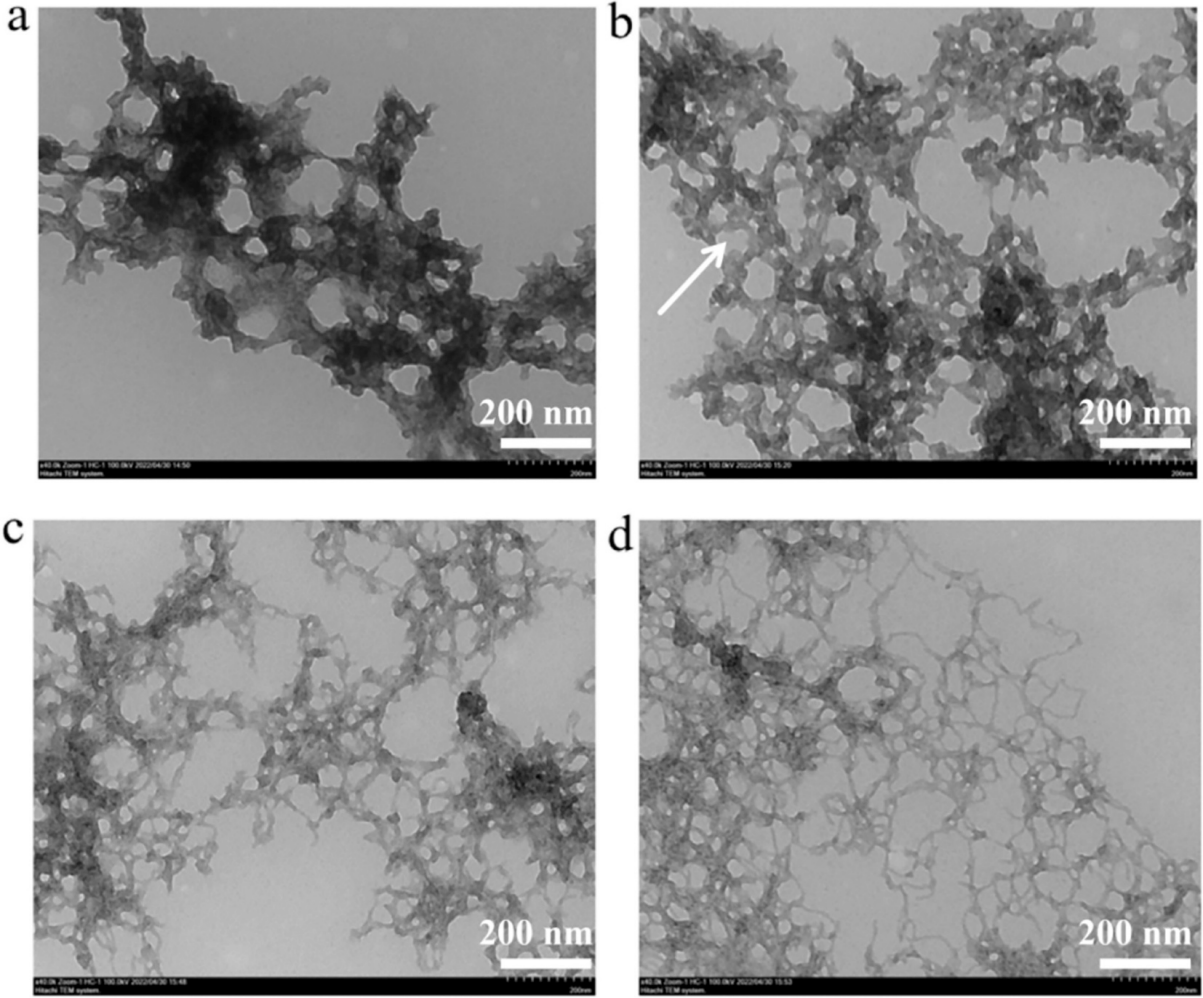
TEM images of MPNFs formation during acid-heat treatment (pH 2.0, 90 °C) at different time points: (a) 0 h, showing globular MPI aggregates; (b) 0.5 h, with emerging protofibrils (arrows); (c) 6 h, elongated fibrils forming networks; (d) 11 h, mature worm-like MPNFs with an entangled network. Samples were negatively stained with 2% uranyl acetate. Scale bars represent 200 nm.

TEM images delineated the structural evolution during MPNFs formation (Fig. 1). The native MPI primarily existed as compact macromolecular aggregates (Fig. 1a). As the self-assembly proceeded, these aggregates underwent structural rearrangement, yielding looser clusters with emerging slender and curved protofibrils (Fig. 1b, c). Upon maturation (11 h incubation), a well-defined, intertwined network of worm-like fibrils was established (Fig. 1d), consistent with the morphology reported for certain plant-derived PNFs (Herneke et al., 2023). This distinct worm-like architecture, contrasting with the straight fibrils typical of many animal proteins (Liu et al., 2024; Norouzi, Hesarinejad, Kadkhodaee, Nishinari, & Gao, 2026), suggests a different self-assembly pathway and underpins the unique functional properties observed subsequently.

The mature MPNFs exhibited an average length of 1.2 ± 0.4 μm and diameter of 8.5 ± 2.1 nm (*n* = 100), as measured from TEM images using ImageJ software. These dimensions are comparable to previously reported values for mung bean protein fibrils (lengths of 0.8–1.5 μm and diameters of 5–10 nm)(Qi et al., 2024). Notably, the worm-like morphology of MPNFs contrasts with the straight, rigid fibrils typically observed for whey protein and egg white protein (Qi et al., 2024), suggesting a distinct self-assembly pathway for mung bean 8S globulin. This curved architecture may offer advantages for network formation, as the increased flexibility could facilitate entanglements and junction zone formation during gelation (Li et al., 2025).、.

The structural evolution observed by TEM can be explained by the synergistic effects of acid and heat treatment during fibrillation. At pH 2.0, the net positive charge on MPI increases due to protonation of carboxyl and amino groups, leading to strong electrostatic repulsion that partially unfolds the globular structure and exposes hidden hydrophobic regions. Subsequent heating to 90 °C provides the kinetic energy required for peptide bond hydrolysis and for the alignment of unfolded polypeptide chains into ordered *β*-sheet-rich fibrils. This process is consistent with the nucleation-dependent polymerization mechanism commonly reported for food protein nanofibrils (Cao, Tian, Wang, Wang, & Liu, 2026). The appearance of curved protofibrils at 0.5 h (Fig. 1b) and elongated fibrils at 6 h (Fig. 1c) indicates that fibrillation proceeds through lateral association and elongation. The mature worm-like morphology at 11 h (Fig. 1d) likely reflects the specific amino acid sequence and domain structure of mung bean 8S globulin, which may favor a flexible, curved architecture over the straight fibrils typical of whey protein (Chen et al., 2026; Khalid et al., 2025). This curved shape could provide more entanglement points, contributing to the enhanced gel network formation observed in subsequent experiments.

Based on the TEM characterization, the mature MPNFs formed after 11 h of incubation (Fig. 1d) were selected for all subsequent emulsion gel preparation and characterization studies. This time point represents the completion of the fibrillation process, yielding a dense network of well-defined fibrils with consistent morphology and maximum aspect ratio, which are expected to provide optimal functionality for emulsification and gel network formation.

### 3.2 *Effect of* different *preparation conditions on* emulsion *gel formation*

The addition of ions (normally Ca^2+^ in the form of CaCl_2_) can promote soluble protein aggregates to form a gel network by ionic cross-linking (Li et al., 2025). The emulsion and emulsion gels were prepared with MPI as the matrix, and the obtained emulsion demonstrated fluidity under different oil-phase fractions (Fig. S1a upper layer). The results of the MPI emulsion gels formed by Ca^2+^-induced cross-linking of the MPI emulsion are presented in the lower panel of Fig. S1b lower layer. When the oil-phase fraction was 0.25, no emulsion gels were formed. Similarly, when the oil-phase fraction was 0.5, Ca^2+^ at a concentration of 50 mM (abbreviated as emulsion gel 5%P-0.5-50, the same as below) did not induce emulsion gel formation. However, at a Ca^2+^ concentration of 100 mM, the emulsion gels were successfully prepared. Finally, when the oil-phase fraction was 0.75, both 50 and 100 mM Ca^2+^ induced gel formation. The presence of Ca^2+^ facilitated cross-linking and stabilized the emulsion structure, ultimately leading to gel formation. The superior emulsification and gelling capacity exhibited by MPNFs relative to MPI may be attributed to the grid-like structure formed by the nanofibrils.

**Fig. 2:**
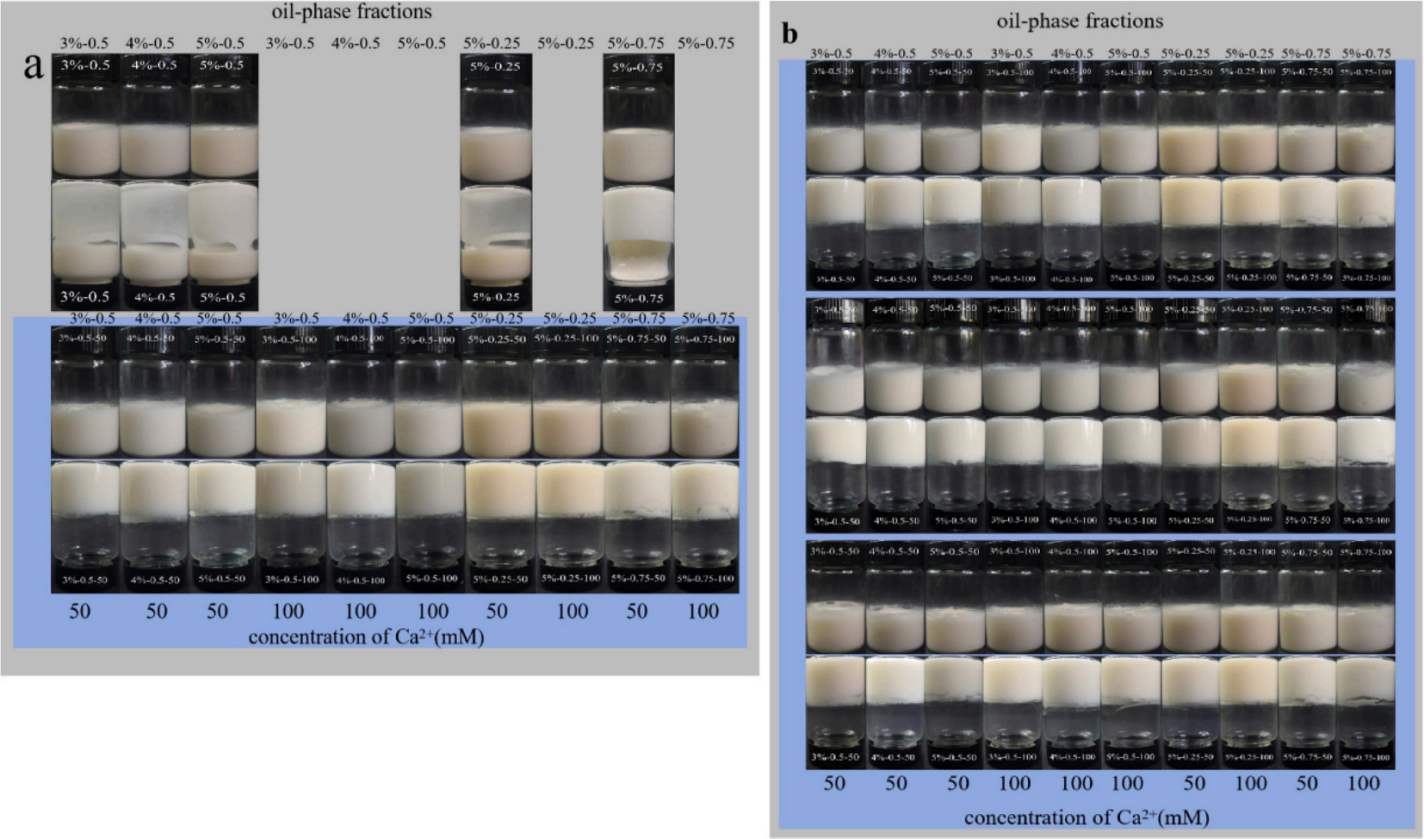
(a) Formation of MPNFs emulsion not crosslinked with calcium ions (upper layer) and emulsion gel crosslinked with calcium ions (lower layer); (b) storage stability of MPNFs emulsion gels after 1, 30, and 60 days at room temperature (from top to bottom), and storage stability.

The emulsions prepared with MPNFs were shown in Fig. 2 upper layer, which demonstrated fluidity that is, as the oil-phase fraction increased, the emulsion consistency increased and fluidity decreased. The emulsion gels prepared with different MPNFs emulsions by Ca^2+^-induced cross-linking were shown in the lower panel of Fig. 2 lower layer, and the emulsion gels were successfully prepared under all conditions. Compared with the results of MPI emulsion gel preparation, the successful formation of emulsion gels by MPNFs under conditions where MPI failed to form emulsion gels indicated that the MPNFs emulsions were better at forming gels under Ca^2+^ induction. For MPI, gelation required ≥100 mM Ca^2+^ and oil fraction ≥0.5; for MPNFs, stable gels formed even at 50 mM Ca^2+^ and oil fraction 0.25. The successful formation of MPNFs emulsion gels under all tested conditions, in stark contrast to the limited gelation of MPI emulsions, unequivocally demonstrates the superior emulsifying and gelling capabilities of MPNFs. This enhancement is attributed to the distinctive worm-like fibrillar network of MPNFs, which provides a larger surface area and more interaction sites for effective Ca^2+^-mediated cross-linking and oil droplet stabilization. The network structure was formed by fibrils aggregates through chemical forces such as electrostatic interaction, hydrophobic interaction, hydrogen bond, and ionic bond (Chen et al., 2026; Xiao et al., 2025), which led to 3D structure with improved gel performance. This conclusion is consistent with the findings of Yang et al. (Yang et al., 2022).

### 3.3 Storage stability of emulsion gels

The successfully prepared MPI and MPNFs emulsion gels were stored at room temperature. The photos of the samples were taken after being stored for 1, 30, and 60 days, and were shown in Figs. S1b and 2b. Both the MPI and MPNFs emulsion gels did not change significantly after 1, 30, and 60 days of storage, showing good storage stability.

Therefore, MPI has been proven to be unsuitable as a base material, and all subsequent in-depth research will focus on MPNFs-based gels. Furthermore, all successfully formed MPNFs gels exhibit good macroscopic stability.

### 3.4 Determination of WHC of emulsion gels

From the macroscopic images of MPI emulsion gels (Fig. S1a), it can be observed that 5%P-0.25-50, 5%P-0.25-100, 5%P-0.5-50, and 5%P-0.5-100 failed to form emulsion gels. Therefore, the MPI samples that failed to gel were excluded from further characterization. All subsequent analyses focus on the successfully formed gels.

**Fig. 3:**
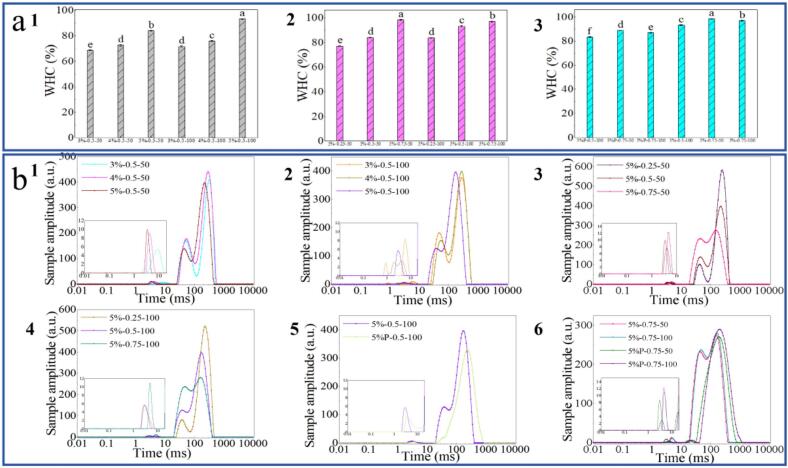
(a) Determination of the water-holding capacity of emulsion gels; (b) transverse relaxation time distribution of emulsion gels.

The WHC of different emulsion gels was measured (Fig. 3). The emulsion gel lost less oil and water when centrifuging, because the gel structure had stronger WHC, showing better structural stability. Fig. 3a1 shows that under the same oil phase fraction and Ca^2+^ concentration, the WHC of the emulsion gels increased with the increase in MPNFs concentration. This indicates that under Ca^2+^ induction, emulsion gels of three different concentrations of MPNFs can be made denser and more stable, with the fibrils at a 5% concentration being the most stable.

Under the condition of the same MPNFs and Ca^2+^ concentrations, emulsion gels show better WHC, the higher the oil-phase fraction, the better the WHC of the emulsion gels (Fig. 3a2). The droplet size of MPNFs emulsion was positively correlated with the oil-fraction, and the structural stability formed under the induction of Ca^2+^ was also positively correlated with the oil phase fraction. Based on the analysis of Fig. 3a2, when the oil-phase fraction was 0.25 and 0.5, the WHC of the emulsion gels increased with the increase in Ca^2+^ concentration, indicating that 100 mM Ca^2+^ induced a more stable gel structure than 50 mM Ca^2+^, and when the oil-phase fraction was 0.75, the WHC of the emulsion gels induced by 50 mM Ca^2+^ was higher than that of 100 mM Ca^2+^. This was because under high oil phase fraction, emulsion droplets were small in size and large in number, which will form a dense gel network structure under Ca^2+^ induced cross-linking, which made the gel structure difficult to lose water, and its water retention was as high as 98.45% (5% -0.75-50) and 97.05% (5% -0.75-100). However, excessive concentration of Ca^2+^ could induce numerous emulsion droplets cross-linking to form a granular gel network structure. Compared with the fine-chain gel network induced by low-ion concentrations, the structural stability of emulsion was reduced, and the WHC deteriorated (Liu et al., 2023). Lower oil phase fraction resulted in smaller emulsion droplets, which means more Ca^2+^ concentration was required.

From Fig. 3a3, it can be seen that under the same conditions, the WHC of MPNFs emulsion gels was higher than that of MPI emulsion gels, indicating that MPNFs emulsion gels had better WHC, and its gel structure was more stable. Among all emulsion gel samples, only emulsion gels 5%-0.5–100, 5%-0.75–50, and 5%-0.75–100 had WHC exceeding 90%, which were 93.29%, 98.45%, and 97.05%, respectively, indicating that these three samples have excellent WHC and form a very stable gel network structure. After centrifugation, the gel network structure was still stable, and only a small amount of water and oil precipitated.

### 3.5 Determination of emulsion gels by LF-NMR

In LF-NMR analysis, the transverse relaxation time (T₂) reflects the mobility and distribution of water protons within the gel matrix (Shrestha, Hag, Haritos, & Dhital, 2023). Generally, three distinct populations can be identified: T₂₁ (1–10 ms) represents bound water tightly associated with protein molecules, T₂₂ (10–100 ms) corresponds to immobilized water trapped within the gel network, and T₂₃ (100–1000 ms) represents free water with high mobility. Shorter relaxation times and smaller peak areas indicate stronger water binding and more restricted water mobility, reflecting improved water-holding capacity (Yu Zhang et al., 2020).

The emulsion gel sample was subjected to low-field nuclear magnetic resonance measurement, and the results are shown in Fig. 3b and Tables S1–S3. As shown in Fig. 3b1, 2 and Table S1, the T_23_ of the emulsion gels with the same oil-phase fractions and Ca^2+^ concentrations decrease significantly with the increase in MPNFs concentration, showing that the emulsion gel with high MPNFs concentrations has stronger WHC. As can be seen in Fig. 3b3, 4 and Table S2, emulsion gels cross-linked at the same MPNFs concentration and concentration, and T_23_ and A_23_ decreased with the increase in oil-phase fraction, indicating that the emulsion gel formed by high oil-phase fraction has a more stable structure and can better stabilize water in the gel network. As presented in Fig. 3b5, 6 and Table S3, at the same oil phase fraction and Ca^2+^concentration, the T_23_ and A_23_ of the emulsion gels prepared by MPNFs were lower than those prepared by MPI, indicating that the MPNFs emulsion gel has better WHC and more stable gel network structure. At the same matrix, the same concentration of emulsion gels at the oil-phase fractions of 0.25 and 0.5 and the T_23_ and A_23_ of the emulsion gel induced by 100 mM, Ca^2+^ was lower than the emulsion gel induced by 50 mM Ca^2+^, which showed that the emulsion gel structure induced by 100 mM Ca^2+^ was more stable and had stronger WHC. When the oil-phase fraction was 0.75, 50 mM Ca^2+^ induced to form a more stable emulsion gel, and its T_23_ and A_23_ were lower than 100 mM Ca^2+^ induced emulsion gels, showing that fine-chain gels compared with particle type gels could form a more stable gel network structure. The low-field NMR results are consistent with the WHC results, and emulsion gels 5%-0.5–100, 5%-0.75–50, and 5%-0.75–100 showed more excellent WHC.

### 3.6 Determination of the rheological properties of emulsion gels

Based on the WHC and LF-NMR results, gels crosslinked with 100 mM Ca^2+^ generally exhibited higher stability than those with 50 mM Ca^2+^. Therefore, for the evaluation of fibril concentration and oil-phase fraction, we selected gels crosslinked with 100 mM Ca^2+^. To directly compare the effect of Ca^2+^ concentration, both 50 mM and 100 mM Ca^2+^ crosslinked gels were tested separately.

**Fig. 4:**
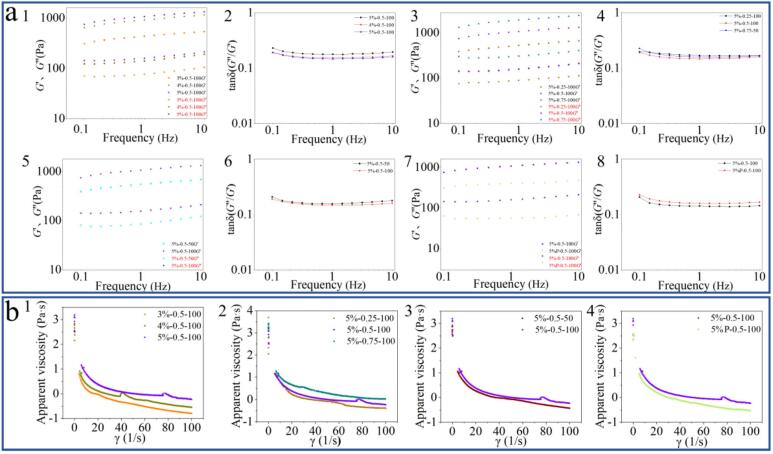
(a) Frequency scanning curves of emulsion gels; (b) variation curves of the apparent viscosity of emulsion gels with shear rates.

Fig. 4 shows the frequency sweep curves of 0.5–100 emulsion gels at different MPNFs concentration, 5%-100 emulsion gels at different oil-phase fraction, 5%-0.5 emulsion gels at different Ca^2+^concentration, 0.5–100 emulsion gels at different water phase. The storage modulus (G′) of all emulsion gel samples in the entire frequency range was higher than that of the loss modulus (*G*″) and exhibited the properties of an elastic emulsion gel. G′ and *G*″ demonstrated little dependence on frequency and did not change greatly with frequency, indicating that the structure of emulsion gel formed by Ca^2+^induced lotion crosslinking was stable. As presented in Fig. 4a1, at 1 Hz, the G′ values for 3%-0.5–100, 4%-0.5–100, and 5%-0.5–100 were 342 Pa, 612 Pa, and 853 Pa, respectively, confirming the concentration-dependent reinforcement of the gel network. The higher the concentration of MPNFs, the higher the G′ and *G*″, with the higher G′ indicating that the gel was more structurally stable and more elastic and less deformable (Zeng, Lee, Jo, & Choi, 2023). High MPNFs concentration was conducive to the formation of emulsion droplets with smaller particle size, which contributed form large and dense aggregates, so the emulsion gel formed by 5% (w/v) MPNFs was more stable. Fig. 4a3 indicated that the higher the G′, the more stable the emulsion gel, that the high oil-phase fraction also affects the size of the emulsion droplets, and a denser gel structure is formed at high oil-phase fractions.

When comparing the effects of Ca^2+^ concentration, the fibrils concentration of 5% and oil phase fraction of 0.5, which yielded the best 3D printing results in the later stage, were selected for testing (Fig 4a5, 6). To compare the influence of protein matrix (MPNFs vs. MPI), two representative formulations (5%-0.5–100 and 5%P-0.5-100) were chosen for comparison (Fig. 4a7, 8).

As illustrated in Fig. 4a5, the emulsion gels induced by 100 mM Ca^2+^ has a higher G′ value than the emulsion gels induced by 50 mM Ca^2+^, indicating that a more stable emulsion gel network structure is formed, and Ca^2+^ concentration induces the emulsion to form a gel. Within the ion concentration range studied, a fine, chain-like gel network was formed; the higher the ion concentration, the more sufficient the reaction, making the gel structure more stable. The effect of Ca^2+^ concentration on emulsion gel strength can be understood at the molecular level. At pH 2.0, the carboxyl groups (–COOH) of glutamic and aspartic acid residues on MPNFs are partially deprotonated, creating negatively charged sites (–COO^−^). When Ca^2+^ are introduced, they act as ionic bridges between two negatively charged carboxylate groups on adjacent fibrils or between fibrils and oil-droplet surfaces. This cross-linking mechanism is often described as an “egg-box” or “ionic bridging" model, similar to that observed in alginate or pectin gels (Liang et al., 2020).

At low Ca^2+^ concentration (50 mM), only a fraction of the available binding sites are occupied, resulting in a fine-chain, flexible network with moderate G′ values. As the Ca^2+^ concentration increases to 100 mM, more ionic bridges form, leading to a denser, stiffer network with higher G′ (Fig. 4a5). This network exhibits enhanced water immobilization (shorter T₂₃ in LF-NMR) and higher WHC (Fig. 3a2). However, when Ca^2+^ becomes excessive (e.g., >100 mM at high oil fraction, as observed in Section 3.4), over-cross-linking can occur, causing the fibrils to aggregate into large, granular clusters rather than a homogeneous network. This granular structure reduces the gel's ability to retain water and oil, explaining the lower WHC at 100 mM Ca^2+^ when the oil fraction is 0.75. Similar dose-dependent behavior has been reported for other protein and polysaccharide systems (Wang et al., 2025).

Fig. 4a7 shows that the G′ of the emulsion gels formed based on MPNFs was higher than that of the emulsion gels formed based on MPI, demonstrating that the better emulsification of MPNFs results in the formation of smaller emulsion droplets that formed a more stable and dense gel network structure due to Ca^2+^ induced cross-linking. Tanδ (*G*″/G′) of the emulsion gels was shown in Fig. 4a2, 4, 6, and 8, which represented the ratio of the lost energy value to the stored energy value and could distinguish gel strength. The tanδ values of all samples remained stable at approximately 0.2 across the frequency range, indicating that the emulsion gels exhibited elastic-dominant behavior (G′ ≫ *G*″). This characteristic is essential for maintaining shape fidelity after extrusion in 3D printing (Manoi & Rizvi, 2009). While yield stress was not directly measured in this study, the successful printing of complex structures (Fig. 6) indirectly indicates that the optimal MPNFs gel possesses sufficient yield stress to resist post-deposition deformation. Quantitative amplitude sweep analysis is planned for future work.

Fig. 4b1, 2, 3, 4 shows the apparent viscosity test results of different emulsion gels. All emulsion gels show a trend of decreasing apparent viscosity with the increase in shear rate, showing shear thinning behavior. Comparison of the maximum viscosity values of the different emulsion gels showed that high concentration of MPNFs (emulsion gel 5%-0.5–100) resulted in higher maximum viscosity value, high oil-phase fraction (emulsion gel 5%-0.75–50) resulted in higher maximum viscosity value, and 5%-0.5–50 had a low maximum viscosity value compared to 5%-0.5–100. The maximum viscosity value of the emulsion gels prepared with MPNFs as matrix under the same conditions (emulsion gel 5%-0.5–100) had a higher maximum viscosity value than the emulsion gel prepared on the MPI matrix (emulsion gel 5%-0.5–100) under the same conditions.

### 3.7 Texture analysis of emulsion gels

According to the above results, the performance of emulsion gels crosslinked with 50 mM Ca^2+^ was inferior to those crosslinked with 100 mM Ca^2+^. Thus, 100 mM Ca^2+^ was fixed in the subsequent experiments. Six representative samples were selected for further characterization and 3D printing:3%-0.5–100, 4%-0.5–100, 5%-0.5–100, 5%-0.25–100, 5%-0.75–100, and 5%P-0.5-100.

The texture analyzer was used to evaluate the physical properties of samples by simulating the human sense of touch and describing them numerically (Nishinari et al., 2024). The hardness of 3D-printed products significantly affects consumer acceptance; therefore, texture testing is essential (Zhou et al., 2025). The texture properties of the emulsion gels were evaluated in terms of hardness, elasticity, cohesion, viscosity, chewiness, and resilience (Table 1).

**Table 1:** TPA values of different emulsion gels.

Sample	Hardness (g)	Springiness	Cohesiveness	Gumminess	Chewiness	Resilience
3%-0.5–100	33.684 ± 0.431f	0.987 ± 0.065e	0.326 ± 0.027f	28.026 ± 0.429f	36.954 ± 0.437f	0.391 ± 0.039e
4%-0.5–100	41.546 ± 0.460e	1.166 ± 0.037d	0.413 ± 0.033e	30.565 ± 0.482e	43.372 ± 0.483e	0.481 ± 0.024d
5%-0.25–100	62.564 ± 0.707c	1.655 ± 0.016c	0.622 ± 0.039c	42.558 ± 0.581c	50.347 ± 0.281c	0.569 ± 0.056c
5%-0.5–100	77.593 ± 0.664b	2.071 ± 0.057b	0.704 ± 0.055b	54.015 ± 0.551b	63.830 ± 0.456b	0.669 ± 0.022b
5%-0.75–100	83.343 ± 0.772a	2.246 ± 0.043a	0.739 ± 0.042a	56.934 ± 0.387a	69.511 ± 0.222a	0.681 ± 0.042a
5%P-0.5-100	58.861 ± 0.606d	1.642 ± 0.046c	0.584 ± 0.035d	38.741 ± 0.346d	47.326 ± 0.561d	0.533 ± 0.063c

**Note**: Different letters in the same column represent significant differences (*p* < 0.05), the same below.

When comparing the texture data of emulsion gels 3%-0.5–100, 4%-0.5–100, and 5%-0.5–100, the concentration of MPNFs increased, and the texture data of the emulsion gels increased, indicating that at high concentrations of MPNFs, the emulsion gel had a more stable structure, was less prone to deformation, and had better texture characteristics. Compared with the texture data of emulsion gels 5%-0.25–100, 5%-0.5–100, and 5%-0.75–100, the oil-phase fraction increased, the texture data of the emulsion gel increased accordingly, and the increase in the oil-phase fraction led to the formation of a denser and more stable emulsion gel network structure, with better texture properties. When comparing the texture data of emulsion gels 5%-0.5–100 and 5%P-0.5-100, the texture data of the emulsion gels revealed that the MPNFs were higher than that of the MPI emulsion gels, indicating that the emulsion gels prepared with MPNFs as a matrix had more excellent texture properties and formed emulsion gels with a more stable structure. In general, emulsion gel 5%-0.75–100 had the best texture properties; however, it was not much different from emulsion gel 5%-0.5–100, and the latter had a lower oil-phase fraction, combined with other tests. As a result, low-fat emulsion gel 5%-0.5–100 was more in line with the concept of a healthy diet when used as a related food base.

### 3.8 Laser confocal image of emulsion gels

**Fig. 5:**
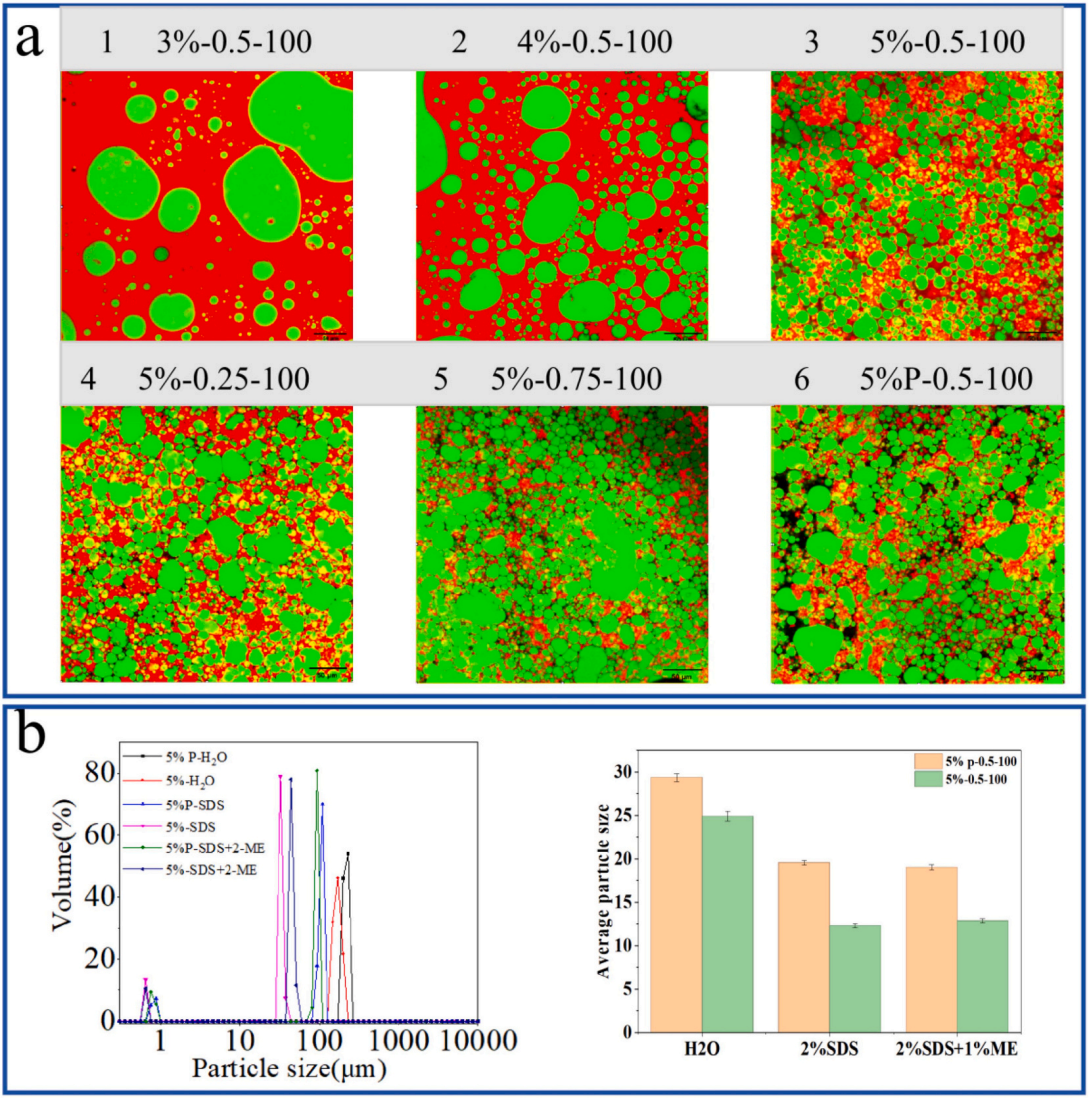
(a) Emulsion gel laser confocal images; (b) particle size distribution of MPI and MPNFs emulsion gels in different dispersants and determination of the average particle size of MPI and MPNFs emulsion gels in different dispersants.

In this experiment, the microstructure of emulsion gels with different MPNFs concentrations (3%, 4%, 5%) and different oil-phase fractions (0.25, 0.5, 0.75) were evaluated by CLSM. A representative MPI-based gel (5%P-0.5-100) was also included for comparison. Nile red was used to dye the oil phase, Nile blue was used to dye MPNFs and MPI, and the results were shown in Fig. 5. By comparing Fig. 5a1–3 the droplet size was found to decrease and the density increase with the increase in MPNFs concentration. Under Ca^2+^ induction, a stable emulsion gel could form with soybean oil at high MPNFs concentrations, exhibiting a more compact and stable network structure.

. Thus, emulsion gel 5%-0.5–100 had better gel properties such as WHC than emulsion gels 3%-0.5–100 and 4%-0.5–100. As shown in Fig. 5a3–5, as the oil-phase fraction increased, the particle size of emulsion droplets decreased while the density increased, forming more stable emulsion gels. This was consistent with Wang's conclusion (Wang et al., 2025) that emulsion gels with high oil phase fraction had good performance. Fig. 5a3 and 5a6 shows the emulsion droplets of MPNFs were smaller and the density was higher; thus, the gel structure of emulsion gel 5%-0.5–100 was more stable than that of emulsion gel 5%P-0.5-100, and it had more excellent gel characteristics. CLSM results clearly and intuitively show the particle size of different emulsion gels, which can verify the results obtained from the subsequent characterization of the particle size determination in emulsion gels.

### 3.9 Particle size determination of emulsion gels

Two representative samples, 5%-0.5–100 and 5%P-0.5-100, were selected for analysis in this section. Particle size is a critical factor that affects the properties of emulsions (Xie et al., 2022). Fig. 5b shows the particle size distribution of MPNFs and MPI emulsion gels in different dispersants. Combined with the average particle size results in Fig. 5b, the influence of emulsion gel particle size in different dispersants and the interaction force between proteins on the surface of emulsion gel droplets can be derived. It was demonstrated that the hydrophobic interaction between proteins and sodium dodecyl sulfate (SDS) was disrupted by the binding of the dodecyl hydrocarbon chain to the hydrophobic region of the protein, which resulted in a reduction in protein particle size (Heidari-Dalfard, Tavasoli, Assadpour, Miller, & Jafari, 2025). Compared with deionized water, the particle size distribution curve shifted significantly to the left after SDS addition, and the average particle size decreased significantly, which indirectly suggests the presence of hydrophobic interactions in the emulsion gel network. The addition of 2-ME to the SDS dispersant caused only marginal further changes, implying that disulfide bonds play a minor role in stabilizing the gel structure. These results indirectly imply that hydrophobic interactions are the dominant forces, while disulfide bonds contribute little, which is consistent with the low cysteine content of mung bean 8S globulin (Qi et al., 2024). The particle size curve of the emulsion gels with SDS as dispersant had obvious bimodal distribution. The addition of mercaptoethanol (2-ME) to the SDS dispersant had no effect on the bimodal distribution, accompanied by a marginal alteration in the particle size profile in comparison to the average particle size dispersed in SDS alone. This indicated that MPI and MPNFs are less affected by the disulfide bond during emulsion gel formation, which is likely due to the absence of disulfide bonds in the main protein 8S globulin in MPI. This also indicates that hydrophobic interactions mainly influenced their formation. Compared with the MPI emulsion gels, the average particle size of the MPNFs emulsion gels was smaller when using different dispersants, which is consistent with the CLSM findings, indicating that the emulsion gel prepared based on MPNFs has better stability.

### 3.10 3D printing

**Fig. 6:**
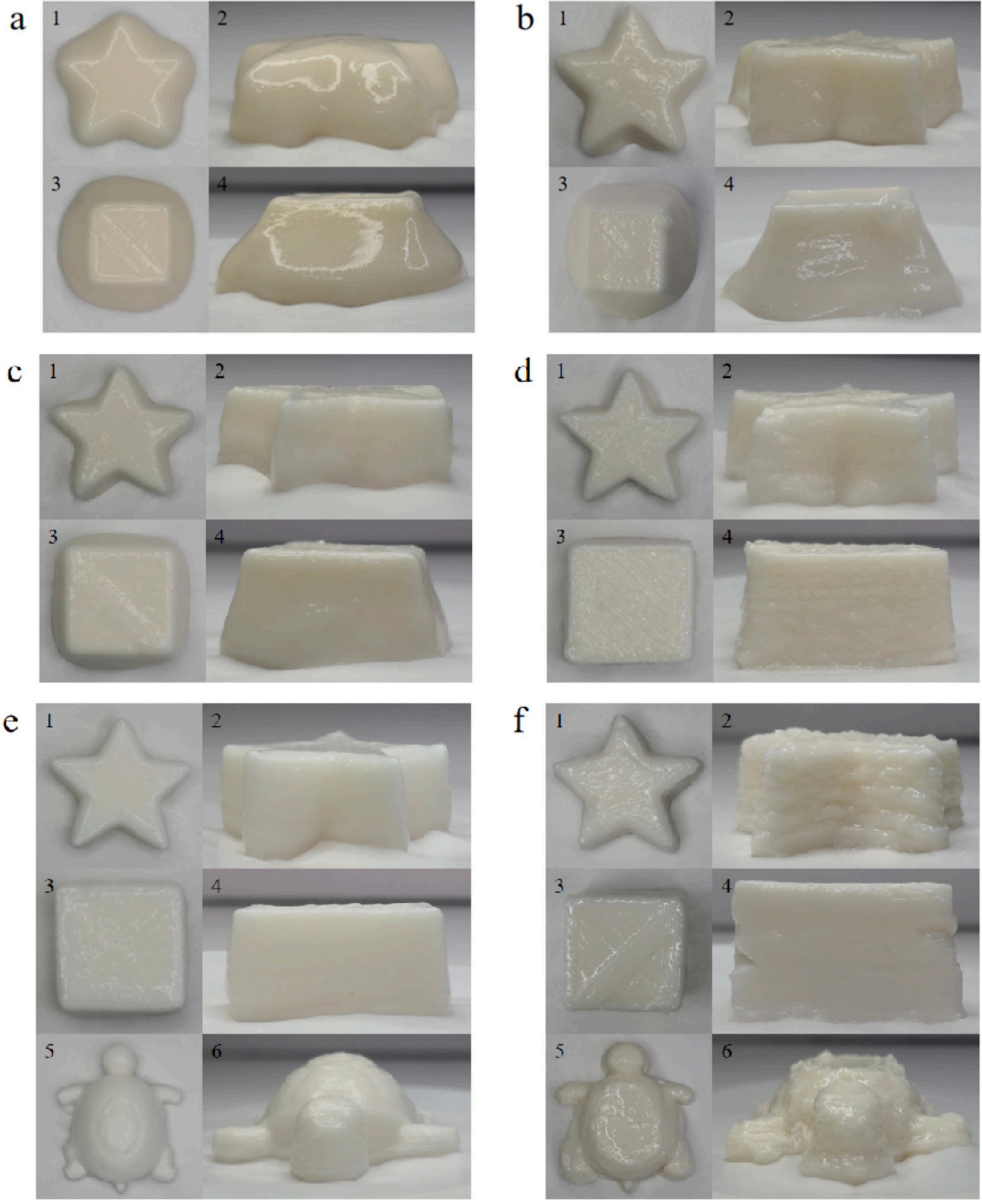
3D printing physical map of emulsion gels (a) 3%-0.5–100, (b) 4%-0.5–100, (c) 5%P-0.5-100, (d) 5%-0.25–100, (e) 5%-0.5–100, and (f) 5%-0.75–50.

3D printing is an additive manufacturing technology and a new food-processing technology (Nie, Wang, Huang, Zhu, & Qin, 2025). 3D printing offers unprecedented design freedom, enabling customization of ingredient composition, shape, and texture according to individual preferences or dietary requirements (Xu, Zheng, Guo, & Chen, 2025). The assessment of 3D printability provided a direct and practical evaluation of the functional performance of the developed emulsion gels. The printing outcomes, as visualized in Fig. 6, revealed a stark contrast between the MPI and MPNFs-based inks, which can be directly correlated with their previously characterized rheological and textural properties.

The MPI-based emulsion gel (5%P-0.5-100) and the low-concentration MPNFs gels (3%- and 4%-0.5–100) exhibited poor shape fidelity. The MPI gel printed cube suffered from significant structural collapse and a stepped appearance (Fig. 6c), while the 3% and 4% MPNFs gels could not maintain the integrity of the printed pentagram and cube, showing severe sagging (Fig. 6a, b). These failures are mechanistically attributed to their insufficient mechanical strength and network cohesion. Specifically:

The MPI gel possessed the lowest storage modulus (G′ ≈ 300 Pa at 1 Hz, Fig. 4g) and texture profile parameters (Hardness: 58.86 g, Springiness: 1.64, Table 1) among the tested samples. Its weaker, less elastic network, originating from the globular protein structure (Fig. 5f), lacked the rigidity to support the weight of overlying layers, leading to progressive deformation.

The 3%- and 4%-0.5–100 MPNFs gels showed a concentration-dependent improvement, yet remained inadequate. Their G′ values (342 Pa and ∼600 Pa respectively, Fig. 4) and hardness (33.68 g and 41.55 g, Table 1) were significantly lower than those of the 5% formulation. This indicates that their gel networks, while formed, were not robust enough to ensure shape retention post-deposition.

In compelling contrast, the MPNFs-based gels at 5% concentration demonstrated exceptional printability. All printed structures (pentagram, cube) exhibited sharp edges and no visible collapse (Fig. 6d–f). This superior performance is a direct manifestation of the optimal rheological and textural profile achieved by the fibrillar network. The 5%-0.5–100 formulation, identified as the optimal ink, combined a high storage modulus (G′ ≈ 850 Pa, Fig. 4) with pronounced shear-thinning behavior (Fig. 4i). This combination ensured that the material was rigid enough to hold its shape after deposition yet fluid enough to be extruded smoothly under shear. Its high hardness (77.59 g) and springiness (2.07, Table 1) further confirmed a network with sufficient elasticity to maintain layer definition and prevent fusion or sagging after deposition.

The successful printing of a highly complex turtle model (Fig. 6e5, e6) with the 5%-0.5–100 gel unequivocally validated its performance. The gel's dense microstructure (Fig. 5d) and high water-holding capacity (93.29%, Fig. 3) contributed to this stability by minimizing syneresis and maintaining network integrity during and after printing.

A comparative analysis with the 5%-0.75–50 gel provided insights into the balance of properties. Although 5%-0.75–50 possessed even higher hardness (83.34 g) and WHC (98.45%), it showed minor shell collapse in the turtle model (Fig. 6f5, f6). This suggests that its network, potentially due to different cross-linking density or droplet packing at high oil fraction, might have a slightly lower elastic recovery or different viscoelastic balance, marginally impairing the fusion between layers in complex overhangs. Therefore, the 5%-0.5–100 formulation was deemed optimal, offering the best synergy of extrudability (shear-thinning), shape retention (high G′), and nutritional profile (lower fat content).

To investigate the retention state of the printed samples, cubes were selected for measurement. The dimensional stability of the printed cubes was quantitatively evaluated by measuring their length (bottom and top), width (bottom and top), and height immediately after printing and after 30 min of storage (Table S4). For the optimal MPNFs emulsion gel (5%-0.5–100), the dimensions remained extremely close to the designed values (20 mm) after 30 min: bottom length 20.2 ± 0.3 mm (initial 20.0 ± 0.1 mm), top length 20.1 ± 0.1 mm (initial 19.9 ± 0.2 mm), bottom width 20.2 ± 0.2 mm (initial 20.1 ± 0.1 mm), top width 20.1 ± 0.2 mm (initial 20.1 ± 0.5 mm), and height 19.9 ± 0.1 mm (initial 20.0 ± 0.2 mm). The height retention ratio after 30 min was 99.5%, demonstrating excellent shape stability without visible collapse or deformation. In contrast, the 3%-0.5–100 and 4%-0.5–100 gels showed pronounced dimensional deviations, with bottom lengths increasing to 28.6 ± 1.2 mm and 26.4 ± 1.1 mm, respectively, and heights decreasing to 16.3 ± 0.3 mm and 18.4 ± 0.4 mm, indicating severe spreading and collapse. The MPI-based gel (5%P-0.5-100) also exhibited poor stability, with height dropping from 18.9 ± 0.5 mm to 16.4 ± 0.4 mm and bottom width expanding to 26.7 ± 0.8 mm. The 5%-0.75–50 gel showed moderate shape distortion, with height decreasing to 18.7 ± 0.4 mm and bottom width increasing to 21.3 ± 0.3 mm after 30 min. These results confirm that the 5%-0.5–100 emulsion gel possesses superior structural integrity and is suitable for high-fidelity 3D food printing.

This direct linkage between the fibril-induced microstructure, the macroscopic viscoelastic properties, and the final 3D printing outcome conclusively demonstrates that the MPNFs-based emulsion gel is not merely a printable material, but a tailored system whose printability is a predictable outcome of its engineered rheology and texture.

## 4 Conclusions

This study demonstrates that the structural transformation of native MPI into worm-like MPNFs via acid-heat treatment is an effective and feasible strategy for creating high-performance, plant-based emulsion gels tailored for advanced food manufacturing. The acid-thermal induction produced MPNFs with a characteristic worm-like morphology, which served as superior building blocks for a calcium-crosslinked network. This structural shift from globular aggregates to an anisotropic fibrillar network was the cornerstone of the dramatically enhanced functionality.

The resulting MPNFs emulsion gel exhibited a synergistic combination of properties essential for 3D printing: a dense and uniform microstructure, a substantial enhancement in water-holding capacity, and viscoelastic solid behavior with strong shear-thinning. These attributes collectively enabled the precise fabrication of complex 3D structures with exceptional shape fidelity and stability, a feat unattainable with its native MPI counterpart. The comparative analysis unequivocally positions MPNFs as a far more effective emulsifier and gelling agent than MPI.

Beyond demonstrating a novel 3D printing ink, this work provides deeper scientific insight: it establishes protein nanofibrillation as a critical processing step to unlock the latent functional potential of plant proteins. By establishing a clear structure-function relationship, we provide a foundational framework for the rational design of edible colloidal materials. Future work will focus on optimizing the fibrillation process and exploring the incorporation of bioactive compounds within this fibrillar gel matrix. This research paves the way for a new class of sustainable, nutritious, and customizable foods engineered at the microstructural level.

## CRediT authorship contribution statement

**Yupeng Qi:** Writing – original draft, Methodology, Data curation, Conceptualization. **Yuhe Wang:** Writing – original draft, Data curation, Conceptualization. **Chunhong Liu:** Software, Funding acquisition. **Dongmei Li:** Formal analysis, Data curation. **Zhibiao Feng:** Writing – review & editing, Project administration. **Bin Jiang:** Supervision, Project administration, Conceptualization. **Jing Xu:** Writing – review & editing, Software, Project administration.

## Declaration of competing interest

The authors declare that they have no known competing financial interests or personal relationships that could have appeared to influence the work reported in this paper.

## Data Availability

Data will be made available on request.
