# Supplementary material for: Fabrication and characterization of a calcium-induced mung bean protein nanofibrils emulsion gel with tailored rheology for 3D food printing
Source: Food Chem X. 2026 Jul 25;38:104246. doi: 10.1016/j.fochx.2026.104246 (PMC13446037; doi:10.1016/j.fochx.2026.104246)
Supplement: Supplementary file 1 [file mmc1.docx]

**Fig. S1a**:MPI emulsion not crosslinked with calcium ions (upper layer), emulsion gel crosslinked with calcium ions (lower layer) ; **b**: Storage stability of MPI emulsion gels after 1, 30, and 60 days at room temperature (from top to bottom).and storage stability.

**Table S1** Transverse relaxation time and relaxation peak area of MPNFs emulsion gels with different fibrils concentrations

|  | 3%-0.5-50 | 4%-0.5-50 | 5%-0.5-50 | 3%-0.5-100 | 4%-0.5-100 | 5%-0.5-100 |
| --- | --- | --- | --- | --- | --- | --- |
| T_21_(ms) | 2.583/9.011 | 4.119 | 3.409 | 0.912/5.941 | 1.956/4.501 | 2.967 |
| T_22_(ms) | 51.114 | 54.789 | 44.488 | 47.686 | 54.789 | 38.720 |
| T_23_(ms) | 310.787 | 289.942 | 219.639 | 289.942 | 270.496 | 178.343 |
| T_24_(ms) | -- | -- | -- | -- | -- | -- |
| A_21_ | 4.933/69.82 | 65.061 | 54.472 | 11.644/55.531 | 25.164/31.251 | 45.485 |
| A_22_ | 2303.345 | 2394.876 | 1705.359 | 2560.226 | 2126.985 | 1499.198 |
| A_23_ | 4758.916 | 6167.751 | 6005.639 | 5707.059 | 6163.206 | 6757.423 |
| A_24_ | -- | -- | -- | -- | -- | -- |

Note: "--" means not detected, the same below

**Table S2** Transverse relaxation time and relaxation peak area of MPNFs emulsion gel with different oil phase fractions

|  | 5%-0.25-50 | 5%-0.5-50 | 5%-0.75-50 | 5%-0.25-100 | 5%-0.5-100 | 5%-0.75-100 |
| --- | --- | --- | --- | --- | --- | --- |
| T_21_(ms) | 3.917 | 3.409 | 4.824 | 2.768 | 2.967 | 4.824 |
| T_22_(ms) | 41.504 | 44.488 | 44.488 | 38.720 | 38.720 | 47.686 |
| T_23_(ms) | 252.354 | 219.639 | 155.233 | 235.429 | 178.343 | 166.382 |
| T_24_(ms) | -- | -- | -- | -- | -- | -- |
| A_21_ | 52.096 | 54.472 | 64.791 | 54.892 | 45.485 | 51.736 |
| A_22_ | 932.282 | 1705.359 | 3577.428 | 827.563 | 1499.198 | 3756.579 |
| A_23_ | 7444.248 | 6005.639 | 4734.837 | 7524.013 | 6757.423 | 5832.952 |
| A_24_ | -- | -- | -- | -- | -- | -- |

**Table S3** Transverse relaxation time and relaxation peak area of MPI and MPNFs emulsion gels

|  | 5%-0.5-100 | 5%P-0.5-100 | 5%-0.75-50 | 5%-0.75-100 | 5%P-0.75-50 | 5%P-0.75-100 |
| --- | --- | --- | --- | --- | --- | --- |
| T_21_(ms) | 2.967 | 3.654 | 4.824 | 4.824 | 3.181 | 3.917 |
| T_22_(ms) | 38.720 | 12.751 | 44.488 | 47.686 | 18.042 | 22.219 |
| T_23_(ms) | 178.343 | 235.429 | 155.233 | 166.382 | 191.164 | 204.907 |
| T_24_(ms) | -- | -- | -- | -- | -- | -- |
| A_21_ | 45.485 | 39.124 | 64.791 | 51.736 | 40.748 | 16.506 |
| A_22_ | 1499.198 | 8.215 | 3577.428 | 3756.579 | 32.892 | 42.245 |
| A_23_ | 6757.423 | 7338.37 | 4734.837 | 5832.952 | 7285.387 | 7755.864 |
| A_24_ | -- | -- | -- | -- | -- | -- |

**Table S4**. Dimensional changes and shape fidelity of 3D printed cubic models (20 mm × 20 mm × 20 mm) prepared with different emulsion gels immediately after printing (0 min) and after 30 min of storage.

| **group** | **Storage time**  **(min)** | **Length (down)**  **(mm)** | **Length (up)**  **(mm)** | **Width (down)**  **(mm)** | **Width (up)**  **(mm)** | **Height(mm)** |
| --- | --- | --- | --- | --- | --- | --- |
| 3%-0.5-100 | 0 | 25.3 ± 1.0 | 20.7 ± 0.3 | 25.9 ± 0.8 | 20.0 ± 0.4 | 18.4 ± 0.4 |
|  | 30 | 28.6 ± 1.2 | 21.5 ± 0.4 | 28.4 ± 1.4 | 21.7 ± 0.3 | 16.3 ± 0.3 |
| 4%-0.5-100 | 0 | 24.9 ± 0.6 | 19.7 ± 0.4 | 25.3 ± 0.7 | 19.8 ± 0.5 | 19.9 ± 0.3 |
|  | 30 | 26.4 ± 1.1 | 21.8 ± 0.3 | 26.7 ± 0.8 | 21.0 ± 0.3 | 18.4 ± 0.4 |
| 5%P-0.5-100 | 0 | 24.1 ± 0.7 | 18.1 ± 0.3 | 24.8 ± 0.7 | 18.2 ± 0.6 | 18.9 ± 0.5 |
|  | 30 | 26.4 ± 1.1 | 21.8 ± 0.3 | 26.7 ± 0.8 | 21.0 ± 0.3 | 16.4 ± 0.4 |
| 5%-0.25-100 | 0 | 20.1 ± 0.2 | 19.8 ± 0.1 | 19.6 ± 0.2 | 20.1 ± 0.2 | 19.7 ± 0.3 |
|  | 30 | 20.2 ± 0.1 | 20.1 ± 0.1 | 20.2 ± 0.2 | 20.3 ± 0.2 | 19.2 ± 0.5 |
| 5%-0.5-100 | 0 | 20.0 ± 0.1 | 19.9 ± 0.2 | 20.1 ± 0.1 | 20.1 ± 0.5 | 20.0 ± 0.2 |
|  | 30 | 20.2 ± 0.3 | 20.1 ± 0.1 | 20.2 ± 0.2 | 20.1 ± 0.2 | 19.9 ± 0.1 |
| 5%-0.75-50 | 0 | 20.3 ± 0.2 | 19.7 ± 0.2 | 20.2 ± 0.2 | 19.9 ± 0.3 | 19.8 ± 0.3 |
|  | 30 | 21.1 ± 0.4 | 20.4 ± 0.3 | 21.3 ± 0.3 | 20.6 ± 0.3 | 18.7 ± 0.4 |
